# Modeling the public health impact of voxelotor in the management of sickle cell disease in France

**DOI:** 10.1371/journal.pone.0291211

**Published:** 2023-09-13

**Authors:** Frédéric Galacteros, Olivier Ethgen, Maud Beillat

**Affiliations:** 1 Unité des Maladies Génétiques du Globule Rouge, CHU Henri Mondor, Créteil, France; 2 SERFAN Innovation, Namur, Belgium; 3 Department of Public Health, Epidemiology & Health Economics, University of Liège, Liège, Belgium; 4 Pfizer Biopharma Group, Pfizer, Paris, France; University of Illinois at Chicago, UNITED STATES

## Abstract

Sickle cell disease (SCD) is an inherited blood disorder in which sickle hemoglobin (HbS) polymerizes, leading to red blood cell sickling and chronic hemolytic anemia, vaso-occlusive crises, and end-organ damage associated with early mortality. Despite standard of care, patients with SCD still experience complications and early mortality, highlighting remaining unmet treatment needs. Voxelotor is a first-in-class HbS polymerization inhibitor approved by the US Food and Drug Administration as a treatment for SCD and by the European Medicines Agency for hemolytic anemia due to SCD. In clinical studies, voxelotor has been shown to increase hemoglobin (Hb) and decrease hemolytic markers in patients with SCD. The objective of this study was to estimate the impact of voxelotor on the burden of SCD in France using a modeling approach, accounting for its anticipated adoption and diffusion over the next 5 years. We designed a sequential multi-cohort model to project and compare the cumulative incidence of SCD complications over a 20-year time horizon in a world with and without voxelotor. A distribution of patients was simulated across various levels of Hb response based on the phase 3 HOPE trial results, and relative risk reduction was adjusted using published meta-analysis results that projected risk reduction due to a 1 g/dL increase in Hb. In 6100 modeled patients with SCD treated with voxelotor, the model projected the number of deaths to decrease by 39.4%, with an increase of 1.8% in life-years gained. The model also projected life expectancy to increase by 15.8%, and incident cases of stroke, pulmonary hypertension, and chronic kidney disease to decrease by 19.8%, 24.5%, and 25.1%, respectively. The model suggests that improving Hb using a treatment such as voxelotor may have a positive public health impact by reducing the burden of SCD for patients and the healthcare system.

## Introduction

Sickle cell disease (SCD) is a debilitating inherited blood disorder in which abnormal (i.e., sickle) hemoglobin (HbS) polymerizes under conditions of deoxygenation, leading to a cycle of red blood cell (RBC) sickling and unsickling and eventual membrane damage [1]. As a result of this process, patients with SCD experience chronic hemolytic anemia, painful vaso-occlusive crises (VOCs), and numerous acute and chronic complications, including leg ulcers, pulmonary hypertension (PH), and end-organ damage (EOD) [1].

Among therapies approved for SCD in the past few years, voxelotor is a first-in-class HbS polymerization inhibitor that targets the underlying disease mechanism. Voxelotor has been approved by the US Food and Drug Administration for the treatment of SCD in adult and pediatric patients 4 years of age and older and received marketing authorization by the European Medicines Agency as a monotherapy or in combination with hydroxyurea for the treatment of hemolytic anemia due to SCD in patients 12 years of age or older. By binding to the N-terminal α-chain of Hb, voxelotor increases HbS oxygen affinity, thereby inhibiting polymerization that occurs during deoxygenation and potentially leading to reduced RBC sickling, improved RBC health, and prolonged RBC half-life in vitro and in mice [2, 3]. Daily oral administration of voxelotor 900 and 1500 mg was evaluated against placebo in the pivotal phase 3, double-blind, placebo-controlled HOPE study that enrolled patients aged 12 to 64 years [4]. The primary endpoint was the percentage of patients who achieved a Hb response that was defined as a >1.0 g/dL increase in Hb from baseline at week 24. Overall, 51% (95% CI: 41%–61%) of patients treated with voxelotor 1500 mg compared with 7% (95% CI: 1%–12%) of patients who received placebo achieved the pre-specified Hb response at week 24 [4]. Notably, least squares mean change from baseline in Hb level to ≥1 g/dL was achieved as early as week 2 of treatment with voxelotor 1500 mg and was maintained through study end. Additionally, treatment with voxelotor 1500 mg significantly reduced markers of hemolysis (indirect bilirubin level and the percentage of reticulocytes) compared with placebo [4]. Long-term follow-up data confirmed the durable increase in Hb concentration and reduction of hemolysis markers up to week 72 [5].

Although SCD is highly prevalent in sub-Saharan Africa, the Middle East, India, and regions surrounding the Mediterranean, the prevalence of SCD in other regions is growing. In France, the number of people with SCD was estimated to be 19,800 to 32,400 individuals, based on a 2021 analysis that examined SCD prevalence from 2006 to 2016 [6, 7]. Given the recent approval of voxelotor in France, the objective of this analysis was to estimate the possible impact of voxelotor on the burden of SCD in France using a modeling approach, accounting for its anticipated adoption and diffusion pace over the next 5 years.

## Methods

### Analytical model and target population

**Fig 1:**
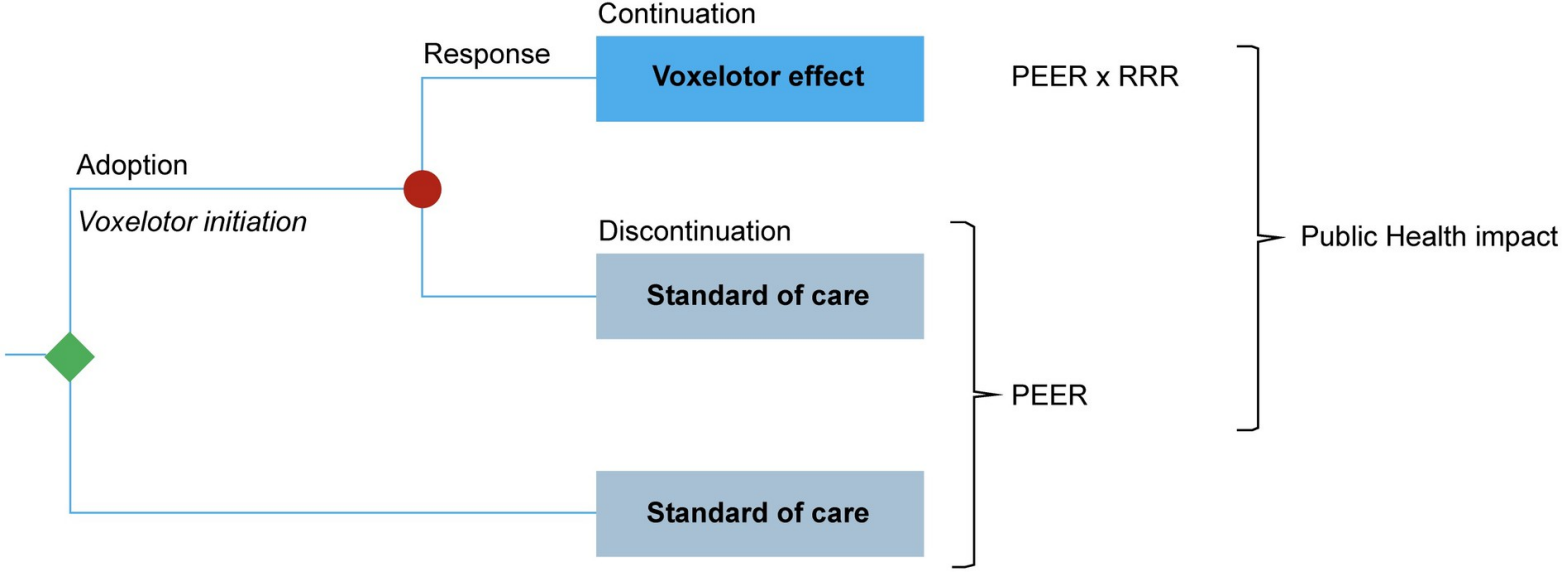
Public health impact model of counterfactual worlds with and without voxelotor. PEER, patient expected event rate; RRR, relative risk reduction.

Using Microsoft Excel, we designed a sequential multi-cohort model of patients with SCD [8, 9] to project and compare the cumulative incidence of SCD complications over a time horizon of 20 years (2023–2043). Two counterfactual states of the world were compared: a world without the availability of voxelotor versus a world with the availability of voxelotor, with all patients in each scenario receiving standard of care (Fig 1). In the world without voxelotor, patient expected event rates (PEER) of SCD complications were applied to all patients. In the world with voxelotor, the same PEERs were applied but adjusted by relative risk reductions (RRR) for patients treated with voxelotor. The RRRs translated the protective effect of an increase in Hb of 1 g/dL on clinical outcomes.

### Treatment adoption and diffusion

The prevalent population and number of incident cases of SCD per year were based on an estimation by the Haute Autorité de Santé that the number of patients with SCD in 2022 who were likely to be treated with voxelotor in France is 5000 to 8000, which was determined by the estimated total population of French patients with SCD [10]. We used a population of 6100 prevalent patients at model inception, adding 300 incident patients each year over the 20-year time horizon (Table 1). We also convened a group of clinical experts in France to discuss and provide input about the patient population with SCD.

**Table 1:** Characteristics of the modeled SCD patient population.

Characteristics	Values
**Epidemiology**	
Prevalent population (2022), N	6100 (4575–7625)
Incident cases per year, n	300 (225–375)
Annual growth rate (of incident cases), %	5.0% (3.8%–6.3%)
**Assumed age distribution**	
12–15 years	15.0% (11.3%–18.8%)
16–24 years	25.0% (18.8%–31.3%)
25+ years	60.0% (45.0%–75.0%)
Overall	100.0%
**Age per age group, mean (SD)**	
12–15 years	13.5 (12.0–15.0)
16–24 years	20.0 (16.0–24.0)
25+ years	33.0 (25.0–41.3)
Overall	26.8
**Life expectancy at birth, years**	55.0 (45.0–65.0)
**Sex, %**	
Female	55.0% (45.0%–65.0%)

Values within round brackets represent lower and upper bounds for the sensitivity analysis.

**Fig 2:**
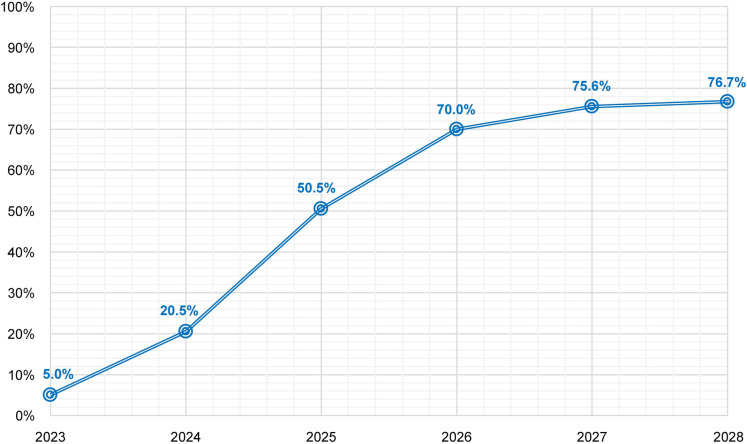
Treatment adoption and diffusion pattern.

We then considered a progressive uptake of voxelotor within the targeted population over 5 years, from 2023 to 2028. Uptake was simulated with a logistic equation depicting a sigmoid adoption and diffusion curve (Fig 2).

Time-to-peak was assumed to be 3 years (ie, in 2026), and the maximum market share at peak was assumed to be 70%—a prevision of treatment coverage at 5 years after launch, which was based on an annual incident growth rate of 5% (Table 1), the lack of availability of comparable treatments, and the estimated number of patients treated annually. This led to an estimated 305, 1308, 3358, 4857, 5473 and 5803 patients to be treated with voxelotor in 2023, 2024, 2025, 2026, 2027 and 2028, respectively.

### Patient expected event rate

PEER [11] was inferred using the lifetime prevalence reported in Johnson et al. 2022, which was used to compute an annualized risk of event based on an assumed life expectancy of 55 years (Table 2) [13]. PEER was then applied to the 2 counterfactual worlds. A limited set of selected complications were included in the analysis: stroke, PH, CKD and death. This selection was guided by the availability of potential RRR from Ataga et al. 2020 [12].

**Table 2:** PEER and RRR associated with an increase in Hb ≥1 g/dL.

Outcomes	PEER^a^	RRR associated with an increase in Hb ≥1 g/dL
		Meta-analysis^b^
**Mortality**		
Death	*—*	0.36 (0.27–0.45)
**Acute crisis**		
Stroke	12.9% (9.7%–16.1%)	0.59 (0.44–0.74)
**Chronic complications**		
PH	49.0% (36.8%–61.3%)	0.43 (0.32–0.54)
CKD	26.6% (20.0%–33.3%)	0.47 (0.35–0.59)

CKD, chronic kidney disease; Hb, hemoglobin; PEER, patient expected event rate; PH, pulmonary hypertension; RRR, relative risk reduction. Values within round brackets represent lower and upper bounds for the sensitivity analysis.

^a^Lifetime prevalence from Johnson et al. 2022 [13].

^b^Ataga et al. 2020 [12].

### Relative risk reduction based on Hb response

**Fig 3:**
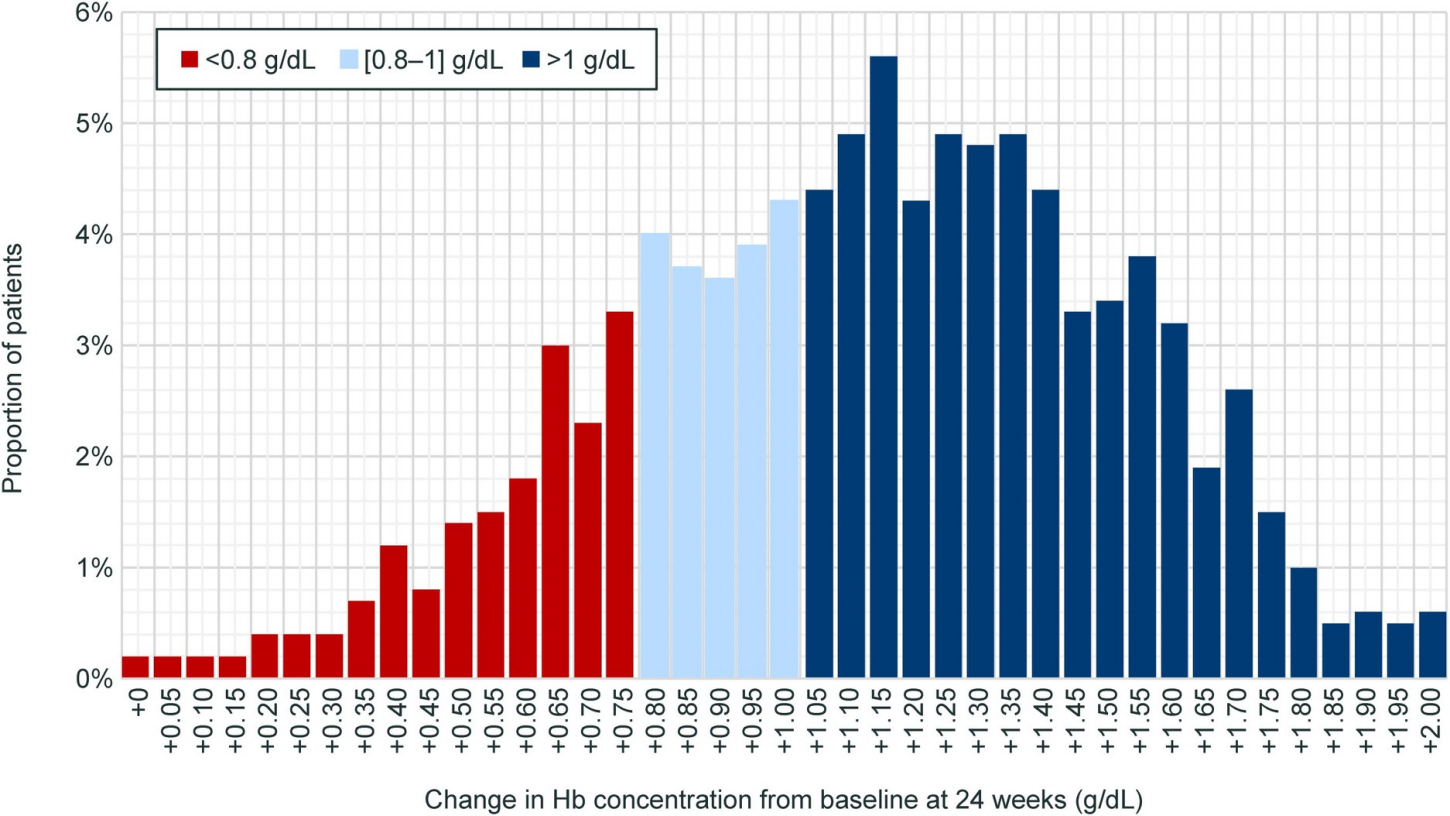
Simulated response distribution of patients across multiple levels of Hb response. Hb, hemoglobin.

A simulated distribution of 1000 patients across various levels of Hb (<0.8 g/dL [18.0%], 0.8–1.0 g/dL [19.5%], and >1.0 g/dL [62.5%]) was developed assuming a normal distribution with a standard deviation (SD) of 0.40 around the mean change from baseline in Hb level reported in the phase 3 HOPE study for the voxelotor 1500 mg group (+1.1 g/dL) at week 24 (Fig 3) [4].

**Fig 4:**
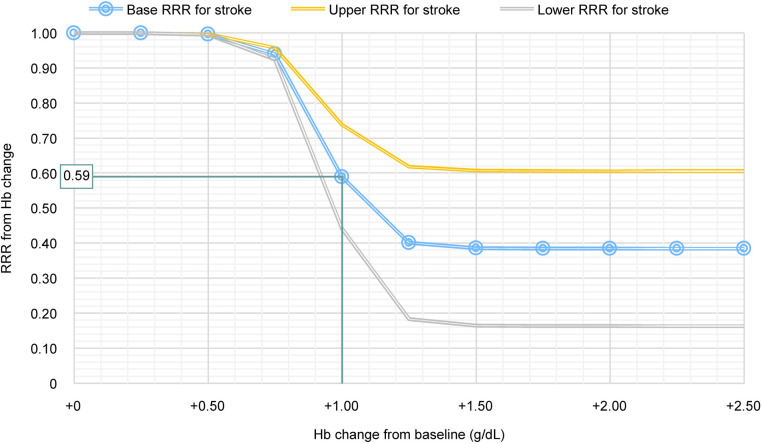
Modeled relationship between Hb response and RRR for stroke. Hb, hemoglobin; RRR, relative risk reduction.

Relative risk reduction was adjusted according to the level of Hb response achieved using a logistic curve and then applied to PEER for those patients who responded to voxelotor (i.e., achieved Hb level >1.0 g/dL), based on the increase in Hb level reported by Ataga et al. (2020) [12]. Patients with an increase in Hb between 0.8 and 1 g/dL were also considered to be “responders” but were modeled with a lower risk reduction than those with an increase >1.0 g/dL. The modeled relationship between Hb response and base RRR for stroke (0.59) is shown in Fig 4; for other outcomes, see supplemental material (S1–S3 Figs).

### Analysis

The time horizon to compute population level outcomes was 20 years (from 2023 to 2043). Using previously determined RRR from the Ataga et al. (2020) meta-analysis, our analysis examined the difference between the 2 counterfactual worlds without and with the availability of voxelotor in France and the impact of Hb response on the reduction of SCD complications, death, life-years gained, and life expectancy [12]. Additionally, one-way deterministic sensitivity analyses were used to identify the top 10 most impactful parameters (with parameter variations within a ±25% range).

## Results

### Target population

Patients were categorized into 3 age groups: 12–15 years (15.0%), 16–24 years (25.0%), and ≥25 years (60.0%). The mean age of patients was 26.8 years, 55.0% were female, and overall life expectancy at birth was 55 years (Table 1).

Over the next 5 years, the percentage of patients treated with voxelotor is predicted to grow from approximately 5.0% in 2023 to 50.5% in 2025 and 76.7% in 2028 (Fig 2). According to the simulated distribution of patients across levels of Hb response (Fig 3), the majority of patients (62.5%) are projected to experience a change in Hb >1 g/dL after initiation of voxelotor treatment. Of the remaining patients, 18.0% are projected to experience a change in Hb <0.8 g/dL, and 19.5% of patients are projected to experience a change between 0.8 and 1.0 g/dL.

### Public health impact of the availability of voxelotor

**Fig 5:**
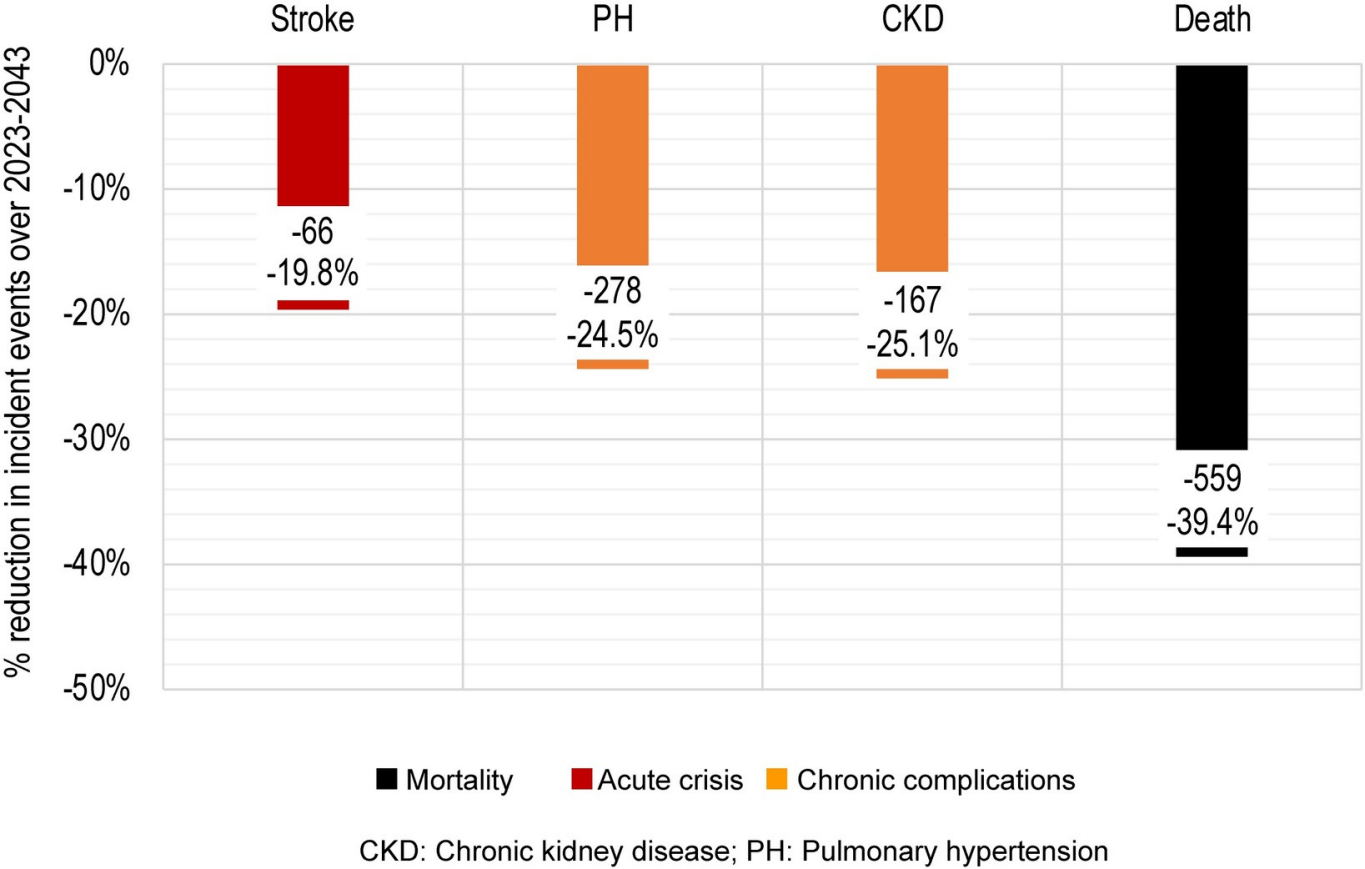
Projected public health impact of voxelotor from 2023 to 2043 according to the RRR associated with an increase in Hb ≥1 g/dL. CKD, chronic kidney disease; Hb, hemoglobin; PH, pulmonary hypertension; RRR, relative risk reduction.

Based on RRR associated with an increase in Hb ≥1 g/dL (Table 3) with voxelotor treatment, the incidences of stroke, PH, CKD, and death are projected to decrease by 19.8%, 24.5%, 25.1%, and 39.4%, respectively, over the next 20 years with the availability of voxelotor (Fig 5).

**Table 3:** Projected public health impact of voxelotor from 2023 to 2043 according to the RRR associated with an increase in Hb ≥1 g/dL^a^.

Outcomes	Impacts according to the RRR associated with an increase in Hb ≥1 g/dL			
	Without voxelotor	With voxelotor	Impact	%
**Survival**				
Number of deaths	1420	861	–559	–39.4%
Life-years^a^	146,225	148,864	+2639	+1.8%
Life expectancy (lifetime)^b^	30.8	35.7	+4.9	+15.8%
**Acute crisis**				
Stroke	334	268	–66	–19.8%
**Chronic complications**				
Prevalent cases (in 2043)				
PH	2773	2030	–743	–26.8%
CKD	1472	1092	–380	–25.8%
Incident cases				
PH	1135	857	–278	–24.5%
CKD	666	499	–167	–25.1%
Life-years spent with complications				
PH	52,957	38,237	–14,720	–27.8%
CKD	27,361	20,054	–7307	–26.7%

CKD, chronic kidney disease; Hb, hemoglobin; PH, pulmonary hypertension; RRR, relative risk reduction.

^a^Life-years were computed using a population approach over a 20-year time horizon (i.e., from 2023 until the year 2043), with the population consisting of six consecutive yearly cohorts of patients.

^b^Ataga et al. 2020 [12].

Over the 20-year time horizon, the number of patient deaths in the world with voxelotor is predicted to be 861 compared with 1420 among patients in the world without voxelotor (Table 3). The impact of voxelotor availability represents 2639 life-years gained and a projected increase in life expectancy of nearly 4.9 years (Table 3). Similarly, the impact of voxelotor availability on the chronic complications of PH and CKD is projected to reduce the number of life-years spent living with either complication by 27.8% and 26.7%, respectively. Lastly, the number of strokes in a world with voxelotor was predicted to decrease by 19.8%, from 334 to 268 (Table 3).

### Sensitivity analyses

**Fig 6:**
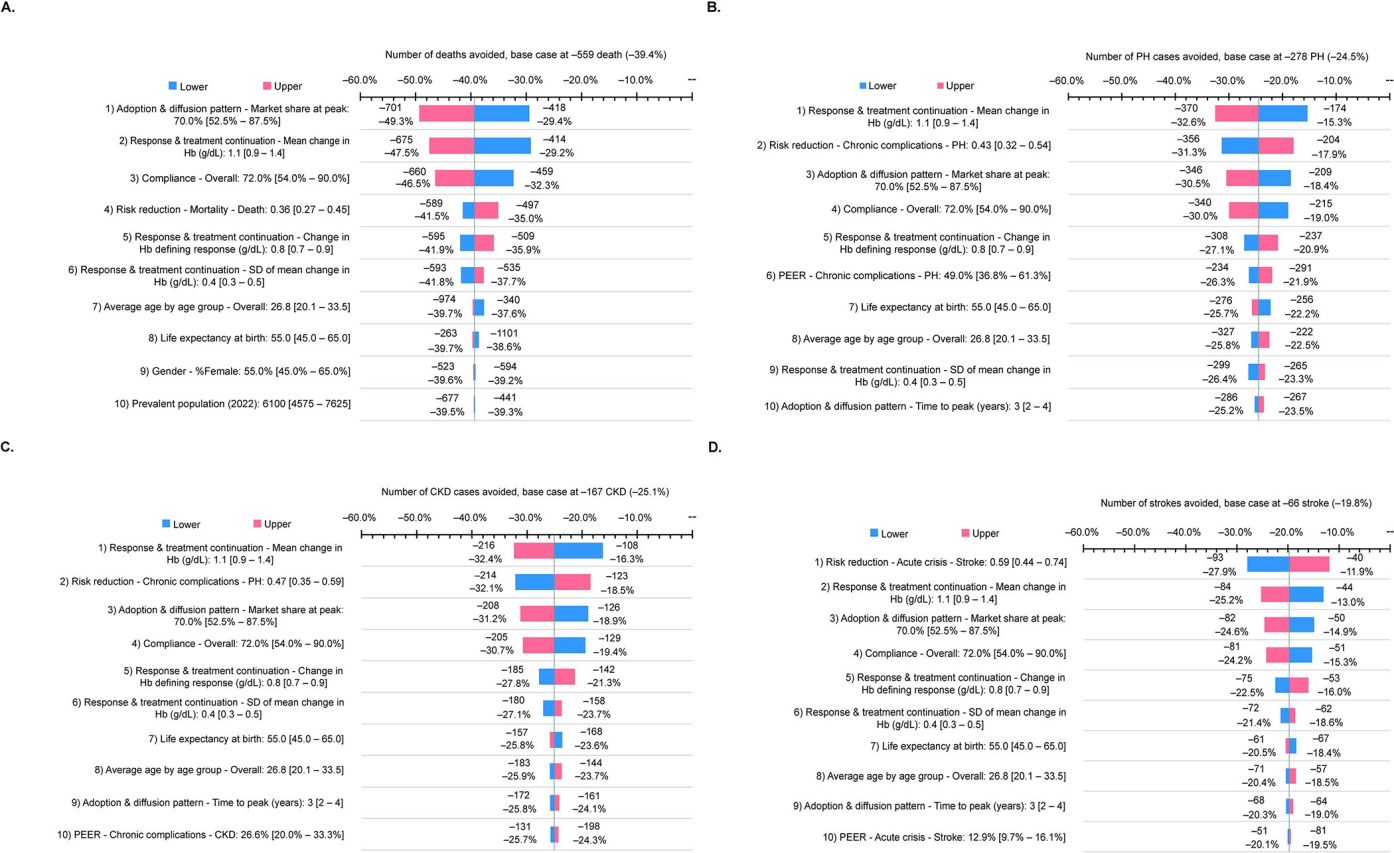
Deterministic sensitivity analysis on selected public health impact metrics. CKD, chronic kidney disease; Hb, hemoglobin; PEER, patient expected event rates; PH, pulmonary hypertension.

A deterministic sensitivity analysis was completed for the predicted number of deaths, PH and CKD cases, and strokes avoided (Fig 6). For predicted deaths avoided, the most influential parameter was the voxelotor adoption and diffusion pattern (market share at peak [range] of 70.0% [52.5%–87.5%], with a range of –49.3% to –29.4% (base case of –39.4%). The second most influential parameter was Hb response and treatment continuation (mean [range] change of 1.1 [0.9–1.4] g/dL), with a range of −47.5% to −29.2% (Fig 6A). For predicted PH and CKD cases avoided, the most influential parameter was Hb response and treatment continuation, with ranges of −32.6% to −15.3% for PH cases avoided (base case of −24.5%) and −32.4% to −16.3% for CKD cases avoided (base case of −25.1%) (Fig 6B and 6C, respectively). The second most influential parameter for PH and CKD cases avoided was RRR (range) associated with an increase in Hb ≥1 g/dL: 0.43 (0.32–0.054) for PH (Fig 6B) and 0.47 (0.35–0.59) for CKD (Fig 6C). For the predicted number of strokes avoided, the most influential parameter was RRR for stroke (0.59 [0.44–0.74]), with a range of −27.9% to −11.9% (base case of –19.8%), followed by Hb response and treatment continuation, with a range of −25.2% to −13.0% (Fig 6D).

## Discussion

We quantified the potential public health impacts of voxelotor on the burden of SCD in France using a modeling approach. To design our sequential multi-cohort model, evidence from a recent risk-reduction meta-analysis of the projected benefits of increased Hb concentrations in patients with SCD was combined with the improvements in Hb levels demonstrated in the phase 3 HOPE trial of voxelotor in patients aged ≥12 years. Based on our findings, including voxelotor as a tool for the treatment of hemolytic anemia due to sickle cell disease may have the possible added benefit of reducing the risk of certain SCD-related complications.

Our model is the first to project the positive public health impact of an increase in Hb >1 g/dL in patients with SCD. Incident reductions of EOD were within the range of −20% to −30%, and the life expectancy gain was nearly 5 years (+15.8%) over the 20-year time horizon. Based on a one-way deterministic sensitivity analysis, the most influential parameters for avoidance of the number of deaths, PH and CKD cases, and strokes included the adoption and diffusion patterns of voxelotor, Hb levels achieved in response to treatment with voxelotor and voxelotor treatment adherence, as well as RRR estimates for each disease complication. Overall, results from the sensitivity analysis were robust over a broad range of varied model parameters.

Based on the results obtained in clinical trials, primarily the phase 3 HOPE trial, voxelotor has demonstrated efficacy in increasing Hb levels and decreasing hemolytic markers in patients with SCD. Non-significant trends were noted in the long-term follow-up of the 24-week HOPE trial, in which voxelotor-treated patients had fewer complications, such as VOCs; improved health status was reported by clinicians and the patients themselves [5]. However, the treatment period in the long-term analysis was only 72 weeks, highlighting the potential usefulness of modeling to project the potential benefits of voxelotor use over a longer period of time and account for real-world variables that are not present in clinical trials. Our model has suggested that including voxelotor as a treatment option for patients with sickle cell disease may have positive public health impacts in relation to SCD complications.

In addition to the meta-analysis by Ataga et al. 2020, real-world evidence (RWE) studies have demonstrated that increased concentrations of Hb are linked to a reduced risk of EOD in patients with SCD. A study based on Clinical Practice Research Datalink and Hospital Episode Statistics with data from 6964 patients with SCD followed up for nearly 7 years in England supports a significant association between an increase in Hb concentration of 1 g/dL with the reduction in the risk for 6 common EOD events (stroke, leg ulcer, PH, CKD, end-stage renal failure, and acute chest syndrome) [14].

Because SCD is a rare disease in some countries, including France, the impact of a particular long-term treatment on the population can be difficult to ascertain. This has motivated us to perform our modeling study, which has allowed us to project population-level effects without investing in a costly study for which results would not be available for many years. The main challenge was to make the model as reliable as possible, so we chose a flexible, analytical tool that can accommodate new data as they become available, which will ultimately improve the precision of the model. Admittedly, it remains challenging to generate mortality and morbidity data over a 20-year time horizon when disease management is likely to be modified as new compounds are being introduced and as others undoubtedly enter the market. Therefore, our modeling exercise addresses some of the challenges of generating such data and may inform future health policies and data generation strategies.

In addition to patient health benefits, the healthcare system may potentially expect some savings as a result of the availability of voxelotor on disease complications. Avoidance of the debilitating, painful, and life-threatening complications included in our model would potentially reduce healthcare resource utilization and therefore lead to cost savings not only for the healthcare system but for individual patients as well. Such an examination of the potential cost savings with voxelotor was not possible for our study because the price and reimbursement status of voxelotor in France were unknown at the time of the study conception. Our approach could complement standard cost-effectiveness and budget impact models by providing insight into the population-wide benefit that can be expected from the adoption and diffusion of a new technology within its intended patient population, which is not informed by these standard models.

Our work remains a preliminary modeling exercise and as such entails several limitations. First, estimates used to document the model parameters were based on scattered evidence from diverse studies or assumptions. Risk reductions with increases in Hb concentration of 1 g/dL were taken from a meta-analysis that included phase 2, 3, or 4 randomized controlled trials, open-label trials, single-arm trials, prospective or retrospective observational studies, database studies, registry studies, or surveys [12]. In our analysis, the increase in Hb as it relates to risk reduction for specific outcomes was thus largely evidence based, even when causation could not be affirmed per se. It has thus yet to be confirmed that the effect reported in these studies will be realized in the real-world setting in France. Second, we only considered four clinical complications for which a protective effect of an increase in Hb concentration was reported in the literature. Many other important complications might need to be accounted for in future analysis to comprehensively depict the full impact of voxelotor. Third, annualized PEER was inferred from lifetime prevalence estimates with an assumed life expectancy for SCD patients. In the absence of data, the approach we have adopted was the most transparent and reproducible. Finally, in the absence of evidence and without access to patient-level data, we applied RRR to PEER as if hazards were proportional.

## Conclusion

In conclusion, including voxelotor as a treatment for hemolytic anemia has the potential to reduce the burden of SCD for individual patients and the healthcare system. This modeling-based finding is expected to be confirmed with RWE data as voxelotor becomes more widely used to treat patients and as evidence of its potential to ameliorate the damaging effects of acute and chronic SCD complications accrues.

## Supporting information

S1 Fig Modeled relationship between Hb response and RRR for death. Hb, hemoglobin; RRR, relative risk reduction.
(DOCX)

S2 Fig Modeled relationship between H response and RRR for CKD. Hb, hemoglobin; RRR, relative risk reduction.
(DOCX)

S3 Fig Modeled relationship between Hb response and RRR for PH. Hb, hemoglobin; RRR, relative risk reduction.
(DOCX)
