# Supplementary material for: Modeling the public health impact of voxelotor in the management of sickle cell disease in France
Source: PLoS One. 2023 Sep 13;18(9):e0291211. doi: 10.1371/journal.pone.0291211 (PMC10499253; doi:10.1371/journal.pone.0291211)

**S1 Fig. Modeled relationship between Hb response and RRR for death.** Hb, hemoglobin; RRR, relative risk reduction.


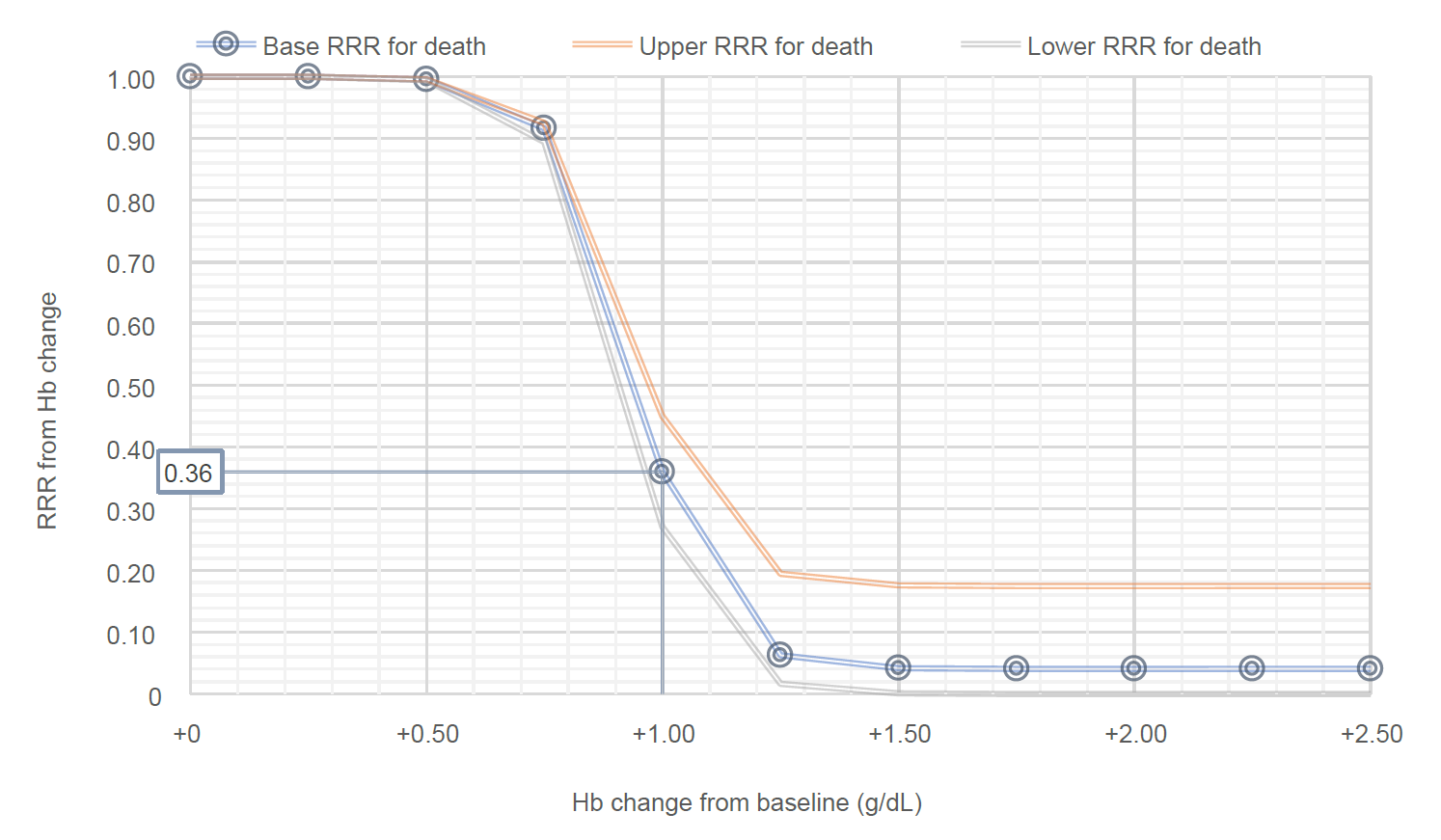

Supplement: S1 Fig — Hb, hemoglobin; RRR, relative risk reduction. (DOCX) [file pone.0291211.s001.docx]
